# Bread, milk and a Tattslotto ticket: the interpretive repertoires of young adult gambling in Australia

**DOI:** 10.1186/s40405-016-0013-0

**Published:** 2016-05-31

**Authors:** Maree Ann Nekich, Keis Ohtsuka

**Affiliations:** Victoria University, Melbourne, Australia

**Keywords:** Young adult gamblers, Discourse analysis, Social gambling, Gambling culture

## Abstract

The discourse of Australian young adults who gamble regularly was analysed to explore key dilemmas and challenges of a generation who grew up with the positive and negative impacts of gambling advertisements. Qualitative interviews of seven young recreational gamblers who regularly frequent gaming machine venues were conducted. The discourse that they used to describe their gambling involvement, motivation, development and subjective views were analysed and three central repertoires: ‘Culture not self,’ ‘If it makes you happy,’ and ‘No problem here!’ were identified. The current findings demonstrate the participants’ attempts to understand and legitimise their gambling. Further, it was suggested that young adults face a series of dilemmas when deciding whether to gamble and to what extent they gamble. Their discourse highlights the tension between individual agency, societal expectations and familial influence. The respondents primarily gambled for social reasons in a manner which they perceived as culturally acceptable. The importance of harm minimization and public awareness campaigns directed at young adults was also discussed.

## Background

 An individual’s intention to engage in behaviour can be predicted from their attitudes, subjective norms and control beliefs (e.g., Ajzen 1991; Ajzen and Madden 1986; Fishbein and Ajzen 1975). Gambling, often regarded as impulsive behaviour, is not an exception (Moore and Ohtsuka 1997, 1999a, b). Young people are more likely to gamble if they have a positive attitude towards gambling, a subjective norm that normalises gambling, and erroneous gambling-related beliefs (EGRBs) (Moore and Ohtsuka 1997, 1999a, b). Although these factors predict gambling behaviour in a general sense, how individuals gamble and regard their gambling is subjective and supported by personally and socially derived meanings attached to the act of gambling. Qualitative research on gambling in the UK has demonstrated the important role of social networks and community activities in the formation of gambling culture (Reith and Dobbie 2011, 2012, 2013). This article aims to further the understanding of gambling in Australia by using discourse analysis to examine the subjective meanings of gambling held by young adult gamblers.

Young adults have a greater propensity for thrill seeking and risk taking compared to older adults. Therefore, it is not surprising that the literature finds the prevalence rate of problem gambling is substantially higher among youth and young adults in many countries. About 18 % of problem gamblers reported their problems with gambling started before the age of 20 years (Productivity Commission 2010). Although not all patrons visiting hotels and bars would gamble, young adults are an important market for the hospitality industry (e.g., Kingma 2004). Since the 1980’s commercial gaming in Australia has grown dramatically. Today’s young adults (18–25 years old) grew up in an environment where easy access to gambling was a norm. About 80 % of Australians gamble at least once a year. The most popular gambling activity is lotteries (participation rate 60 %) while Australians bet the most on “pokies” (electronic gaming machines; EGM) attracting the highest per capita expenditure (Delfabbro 2011). EGMs have been ubiquitous in local bars and social clubs in Victoria since 1992 (Marshall and Baker 2002). Today, 27,633 EGMs are deployed in 530 gaming venues in Victoria and the average annual loss per venue has reached $4,699,063 (Livingston 2013).

In today’s world of instantaneous communication, attitudes and subjective norms are influenced by the information about gambling fed through news media and increasingly social media, making younger people particularly vulnerable due to their high level of connectivity. For example, social media often promote gambling advertisements and online games with gambling themes, some of which require credit purchases to play. Television advertisements of sports betting also target TV viewers around the sports broadcast of professional sporting events (see Gainsbury et al. 2014).

Growing up in a permissive gambling culture, young Australians have positive attitudes towards gambling as an entertainment option, with positive social norms that endorse their gambling participation (Moore and Ohtsuka 1997, 1999a, b). However, harm minimisation and public health messages have largely been dismissed by young adults who do not see the relevance of the warnings to their situation (Hing and Dickerson 2002). Therefore, it is paramount to investigate how young adults conceptualise their involvement in gambling and how this understanding influences current and future behaviour. Exploring their discourse is one way to do this because by focusing on the language they use we can better understand their lived experience of gambling.

Although only a fraction of young people identify that they have a problem with gambling (2–8 %), their prevalence rate is twice as high as that of the adult population. Predictors, antecedents, and correlates of problem gambling include individual characteristics, an early big win or a devastating loss (Rosecrance 1986), cognitive distortions (e.g., Delfabbro and Winefield 2000), illusion of control/impaired control (O’Connor and Dickerson 2003), erroneous statements or gambler’s fallacy (Toneatto et al. 1997), erroneous gambling-related beliefs (Ohtsuka 2015; Ohtsuka and Ejova 2014) and beliefs in fate and destiny (Ohtsuka and Ohtsuka 2010). Further, addictive disorders (Parke and Griffiths 2005), addiction theory (Peele 2001) and personality disorders (Blaszczynski and Steel 1998) suggest genetic predispositions for addiction (Blaszczynski et al. 2005; Reith 1999).

Research literature makes it abundantly clear that gambling is not motivated merely by the pursuit of financial gain or the desire to beat the system (Salkovskis 1996). Otherwise, why would gamblers continue playing despite repeated losses? Nor is the aim of gambling to beat the system, as they continue to gamble after they have won (Reith 1999). The cultural and historical analysis of language use and its influence on gambling through discourse analysis would further address the gap in knowledge about subjective views and meanings associated with gambling (Scott and Trethewey 2008).

Young adult gamblers aged between 18 and 25 years are a vulnerable population. They can participate in any commercial gambling activity freely and may exhibit high levels of adventuresomeness and impulsiveness (Gupta and Derevensky 1998) that predict gambling disorders, yet we do not know much about what problem gambling looks like within this population. Young adults have life goals, lifestyles, and financial demands different from those of older gamblers. Problem gambling may be more prevalent but not as readily visible because they have relatively large disposable incomes, little family or financial obligations, and lack financial assets of significance. However, the negative impact on their personal life is still significant^1^ (Moore and Ohtsuka 1997, 1999a, b). The commonly described gambling harms such as a marriage breakup or a mortgage foreclosure are unlikely to be relevant to young adults. Although warnings are most effective when they are personal, relevant and immediate, there is a deficit in the area of warnings targeting young adults (Mills 2002).

Further, difficult childhood experiences predict addictions including problem gambling (e.g., Jacobs 1988) and gambling behaviour learned early in life tends to be repeated (Sullivan 2001). Converging evidence suggests that young adult gambling is an important phase of gambling history that needs further investigation. In the current investigation, the aim of the research was to investigate how young adults understand and legitimise their involvement in gambling. The findings will contribute towards the creation of effective targeted harm minimisation strategies directed at young adult gamblers to minimise potential gambling harm (Rossen 2001).

Discourse analysis was utilised as a way to explore the language that participants used to describe gambling and their involvement because of its focus on language as the *producer* of phenomena. Hence, the current investigation views language usage not as a neutral medium to describe individual accounts of a phenomenon but emphasises the *constructive* power of the language use and the origin of language in conversations and cultural traditions. If we perceive reality in this way, as constructed and interpreted through discourse and social interaction, then discourse can be understood as the primary organising principle in the construction of reality (Potter 1996).

When a person expresses an opinion or describes an event, the speaker does not merely describe retrieved information but uses their words to construct the meaning actively. When their accounts are constructed, it is highly context-specific because the interlocutors perform a wide variety of social actions (Edley 2001). Discourse is hence a set of “meanings, metaphors, representations, images, and stories, etc. which is used within a culture and shaped by history. It constructs objects, supports institutions and has ideological effects” (Burr 2003). Through discourse, multiple versions of the single event, interpretive repertoires, arise, which may often conflict each other.

### Interpretive repertoires

Interpretive repertoires, derived from ethnomethodology, speech act theory, and semiology, describe a way in which the world can be articulated to present different versions of reality (Potter and Wetherell 1987; Wetherall 1998). They consist of recurrent systems of discursive terms which the speaker uses to characterise and evaluate actions and events (Edley 2001). The speaker selects the repertoire that reflects its intended function, but it is the choice of repertoire that demonstrates how people construct their accounts to appear factual or serve rhetorical functions (Derek and Stokoe 2004). The most commonly used repertoire is called the dominant discourse. As a speaker in a particular society gives cues, another person with the same background will interpret the cues using the *same* interpretive repertoire and achieve the same understanding. Thus, by reacting and reaffirming the invoked repertoire, interlocutors legitimize and construct a reality. The cues in conversational interactions do not necessarily represent the interlocutor’s personality or behaviour. Rather the interlocutor uses discourse markers to represent a particular repertoire that represents one’s worldview (Harper 2006; Kroger and Wood 1998).

For example, the statement “I gamble alone on *Saturday*” could be used to invoke the repertoire of a *weekend* recreational gambler. When cues of problem gambling become salient, the repertoire of the problem gambler who gambles *alone* will become more prominent. Discourse analyses have been used to investigate a speaker’s subjective experiences for a range of topics such as the appraisal of occupational hazards (Scott and Trethewey 2008), the experiences of sexual abuse among South Asian women (Reavey et al. 2006), and self medication amongst the elderly over 80 years old (Lumme-Sandt et al. 2000).

The present study, therefore, aims to analyse culturally shared interpretive repertoires that were used by the young adult gamblers to describe their gambling behaviour and present themselves as people who gamble to socialise.

## Methods

### Participants

A total of seven young adults (five men, two women) aged from 20 to 25 years took part in the study. They described their cultural identity as Australian. All participants acknowledged that they gamble and all but one admitted to frequenting the Hotel on a weekly basis. Each participant was interviewed once, and the interviews took between 50 and 80 min. The occupations of the participants varied from hospitality, trades to administration. Their education levels ranged from the completion of Year 10 (Secondary School), trade qualification (post-secondary vocational education) to the near completion of a medical degree (postgraduate). Verbatim interview transcription was analysed to identify common themes. The number of participants was determined by data saturation. When new information revealed the same discursive themes, the interviewer upon consultation with the second author deemed the data collection complete.

### Materials and procedure

Ethics approval was obtained from the University Human Research Ethics Committee. Participants were recruited by convenience sampling methods and were gaming patrons at a gaming venue. Participants were recruited by direct contact on the evening in which the interview was conducted. Prospective participants included only those who were gambling when approached or who had been observed by the interviewer to be gambling at some stage during the evening. Interviews were conducted by the first author. All prospective participants who had been approached agreed to be interviewed. Semi-structured individual interviews were conducted in a room adjacent to the public bar in a hotel in a northwestern suburb of Melbourne. The interviews covered the topics in the same order, but participants decided how much they elaborated on each topic (See “Appendix” for interview questions). Themes reported in the research literature as correlates of gambling were discussed: the use of free time, familiarity with gambling, gambling of friends and family circles, stories about gambling, views on luck, fate, destiny, and religion. As recommended by Lumme-Sandt et al. (2000), the participants were encouraged to use their own words.

Interviews were audio-recorded, transcribed and re-read several times. According to the convention of interpretative repertoire analysis, a basic transcription scheme representing the words, features such as corrections and hesitations was used (Smith 1995). Individual summaries of repertoires engaged in were created, through which patterns emerged and were compared across summaries. Through continued evaluation of summaries and validation of patterns with raw material, dominant repertoires emerged. The raw data consisted of seven tape-recorded interviews, which were later transcribed. Only a basic transcription scheme representing the words and major features such as corrections and hesitations was used, a common approach when analysing interpretive repertoires (Smith 1995). Every interview was transcribed, read and re-read several times before a detailed analysis was conducted. The analysis consisting of initial basic themes comprised almost entirely of quotes/raw data. The themes were then organised in the way the speakers constructed their accounts to make them appear factual and the way they used their accounts to serve rhetorical functions (as suggested by Tuominen et al. 2002).

## Findings and discussion

Three culturally dominant repertoires were identified: ‘Culture not self,’ ‘If it makes you happy,’ and ‘No problem here!’ All three were readily accessible to all participants and were taken as ‘truth’, however, ‘Culture not self’ showed more variation in expression.

### Culture not self

Culture is the system of knowledge and practice, both explicit and tacit which is shared by a large number of group members. It is a way of life, a cumulative deposit of knowledge, and a human-made component of environment where developmental experiences such as attachment, behavioural learning take place.

When speaking about gambling, the notion of culture^2^ played a pivotal role as it was the primary way they justified and gave themselves permission to be involved. They emphasised the importance of taking a holistic view of the role of gambling in young adults’ lives. Culture includes the family influence that shaped individual views, interests and gambling activities and practice, the social and community influence that created familiarity and permissive attitude toward gambling, and social activities among friends that established acceptance and connection through gambling.

Gambling was discussed not as a conscious lifestyle choice but as an aspect of daily life. Gambling forms such as Tattslotto, scratch lotteries, and the Melbourne Cup were ingrained in the societal consciousness as so mundane that they did not even constitute a ‘real’ gambling activity.

When I get the paper I always pick up a Tattslotto ticket, do you class that? (Steven, Apprentice Chef, 23 years old)

Dad used to get us kids to pick numbers, and he would play those lines every week, and if we won, we would always win like fifth or fourth division occasionally, he would split it between the four of us. (Tristan, Apprentice Carpenter, 18 years old)

We used to do a family sweep at Melbourne Cup but you only win like ten dollars, we didn’t put the money in, but my dad would just pay it out, nothing big at all. There was the football tipping, but it was the same thing more of competition than gambling, but we did get some money if we came first or second at the end of the season. (Tristan, Apprentice Carpenter, 18 years old)

Gambling was a part of the shared cultural experience, a natural way of life for the participants. It was viewed as a common connection and a shared interest transcending age and socioeconomic divide, a tangible activity to bring people together. Their interests in gambling forms often originated from the family culture and tradition that their parents had established. The gambling form that the participant enjoyed was often their parents’ favourite pastime which was fondly remembered. Since gambling was part of their childhood memories of the family tradition, it invoked a great deal of nostalgia, comfort, and normality.

My Nan plays the pokies, my Pa likes the horses better. 

(Steven, Apprentice Chef, 23 years old)

It’s sorta “in my blood” uncle (is a) jockey, old man professional (punter), grandpa bookie. (Michael, University Student, 18 years old)

Oh well my mum, I suppose, she probably … she loves the pokies too. (Peter, Unemployed, 19 years old)

The community influence and the availability of gambling were frequently mentioned. Gambling became a focus of social interaction, and gambling venues were social places that they frequented. The prevalence of gambling in childhood memories fostered a positive regard and acceptance of gambling.

They (my friends) don’t specifically go down to the pub with the intention of betting, but if we are there, they might put money in the pokies or pick a number. (Kate, Receptionist, 21 years old)

Ah well, places like pubs and clubs don’t have stuff like poker and blackjack or roulette and things like that so it (gambling) doesn’t really happen. But pokies…. They’re at the places I go. (Lisa, University Student, 20 years old)

Moreover, sometimes gambling is a rite of passage.

Mum said, “Wow, you are 18, now let’s go to Crown Casino now and gamble!”

(Lisa, University Student, 20 years old)

Gambling was also described as a *group* experience, activity or pastime where all members of a social group had a shared interest. Being good at gambling increased social standing in the group. Gambling was also a way to maintain social interaction—an excuse for catching up. Gambling as a means of social interaction was described freely without defensiveness or fear of social stigma.

It’s mostly pretty accepted, most people I know and am close to have some sort of link with gambling. Say, though I play basketball and in our basketball team, there is one guy who is in a betting syndicate through work, another guy whose dad is an accountant who follows the horses, and he is always passing on tips to us all. And (I have) a friend whose uncle trains greyhounds, and the rest of us are always out as a group so really what some do, the others (also) do to some degree. (Kate, Receptionist, 21 years old)

Gambling is conceptualised in many different activities to socialise with friends rather than narrowly defined as a pastime to win money.

You get a syndicate together and will go in that but nah, when we play golf instead of a ball you get a partner, and you play for a tattslotto ticket. So if you play well, you get a free chance of winning, it only costs you two bucks or whatever. You know that’s a good way to go, sometimes in the TAB you don’t have to bet but, say put in a dollar and say everyone picks a dog and whoever wins, wins the dollars. That’s a way of gambling, but it’s more of a social aspect and just a good thing to do. It doesn’t cost you much (and) you’re with your friends. (Michael, University Student, 18 years old)

The legitimization of gambling to comply with cultural and group norms is a key feature of this discourse. With family, friends, and in the community, gambling as a group enables the participants to avoid personal responsibility. Hence, it was constructed as a purely positive experience supported by a group norm that helped externalise responsibility and avoid moral questions.

### If it makes you happy

Much of the discourse revolved around defining the rights and responsibilities of young adults in their life cycle and their role in the world. Young adults are held accountable for their own actions but not yet burdened with the trappings of older adulthood such as a family of their own, mortgage and children. It was important for them to mention this so that their decisions and actions were understood in the context of young adulthood where they can afford to pursue individual interests. They articulated their justification for their gambling in two ways: first, by minimising and normalising the role of gambling in their life.

I’m 18 and earn a good wage so can afford to spend a lot of money. I don’t have a lot of expenses. I still live at home, I want to save but my only expense is my phone bill, …, I pay a bit for boarding and stuff, but I have at the moment a bit of money to lose. (Michael, University Student, 18 years old)

Second, by describing the valuable qualities of gambling, they justified their gambling that played a positive role in their lives. Again the negative impact of gambling was de-emphasized because it is not the act of gambling that holds value for them but it is the association with a social function that gambling represents. Thus, speaking about gambling means speaking about their life, not because it dictates their life but because gambling venues are where they find their weekly entertainment and friends, where their friendship is formed and strengthened, and the place for support and the oasis to escape from the pressures of everyday drudgery. Gambling presents a challenge in a protected playground where the outcomes are familiar. If they lose bets, it is not a matter of life and death, just money. They stake a small amount of money as their collateral for a little diversion.

I do it with my friends for the enjoyment and not like I said not to make a fortune but for the enjoyment and I mean there is nothing like…. It is, it does give you a bit of a rush, you put your money on the line and then seeing the thing (horse) that you put your money on, your hard earned money on, comes cross the line first. It’s exciting, and it gives you a sense of satisfaction. I’m not generally really a risk taker in life. (Kate, Receptionist, 21 years old)

Gambling was therefore normalized in their language because gambling and gambling venues were described with such familiarity that they no longer warranted excessive explanation.

Not necessarily a good night out, it’s just like another night out. (Theo, Sales Assistant, 24 years old)

We are here at about once a week; there is a group of us, about six usually, we come most Fridays probably for the past twelve months. It’s the one thing you can always rely on that we will come here on a Friday night. (Steven, Apprentice Chef, 23 years old)

The participants legitimized their gambling by its positive attributes: to bring people together, to provide entertainment, excitement and escape from the mundaneness and responsibility in everyday life. Unlike an oasis for emotionally vulnerable problem gamblers (Loughnan et al. 1999), young gamblers perceive gambling as a focal point for socialisation and an excuse to get together. To them, it is a means of socializing with friends, not the end goal of recreation or a cocoon sheltering them from a stressful life. Our participants were mostly single and either employed or on the way to becoming financially independent and had few worries other than socialising.

Indulgence is a viable option for the carefree with a large dispositional income and few financial obligations. However, the most frequently featured and valued attribute was not always explicitly acknowledged—self confidence, a belief in the correctness of their own choice in the face of bewildering freedom of choice.

The participants felt besieged by people directing them in what to do, when to do it, and how to live their lives. Trying to accommodate others’ wishes, they acknowledged the futility of trying to please everyone. They felt pressure to both gamble and to steer clear of gambling. Acknowledging these tensions, they believed that it should be their individual decision to gamble. However, the participants anticipated criticism for their actions whether they decided to gamble or not. Hence, the participants trusted their own decision to gamble so long as gambling would make them happy.

The repertoire ‘if it makes you happy,’ a moderately rebellious yet defensively positive theme was like a mantra in the interviews.

I like going to a pub drinking and having a bet, that’s what I like doing, other people just punt (but) they don’t drink, other people drink and don’t punt, you just gotta be happy, whatever makes you happy. (Michael, University Student, 18 years old)

I find that I believe that you are put on this earth to enjoy it, like abide by rules, to a certain extent to where everyone is happy, but you have got to make yourself happy. If it is gambling, if that is what makes you happy, do it.

(Peter, Unemployed, 19 years old)

Deciding to gamble, the participants’ language was unwavering and uncritical. What if their judgment is flawed? What if the best option for them does not make them happy now but will in the long term? These possibilities, however, were not fully considered in their discourse.

Yep, have some fun, we are not here for a long time we are here for a good time. (Lisa, University Student, 20 years old)

This particular repertoire sums up the young adult gamblers’ worldview of their stage of life. Gambling is a positive activity but unimportant per se. It derives its value through its associations with entertainment, social interaction, fun, escapism, and by the very fact that is was their own decision.

### No problem here!

Although the participants were recreational gamblers who did not experience negative repercussions of gambling, the image of a problem gambler was prominent as the interviewees elaborated to distance themselves from this idea. They described problem gamblers as older gamblers who visited the gaming venue alone. Problem gambling was a solitary affair that occurred outside the social milieu. Problem gamblers were seen to hurt others emotionally and financially and were oblivious to the odds against them. Therefore, the interviewees structured their discourse to distinguish themselves from the image of problem gambling in two ways: they articulated their behaviour as being opposite to problem gambling behaviour, and criticised problem gamblers for their lack of rational thinking. Firstly, the discourse emphasized differences between “us” and “problem gamblers.” Secondly, the participants defended gambling as a positive wholesome leisure activity because they were concerned about a “negative” image that problem gamblers had created. The discourse that acknowledges the potential risk of problem gambling was endorsed to show that they were not at-risk.

I wouldn’t come here by myself, but if I’m meeting my boyfriend or friends, I will come, but I wouldn’t come here to gamble on the poker machines or TAB by myself. (Lisa, University Student, 20 years old)

So it is budgeted for, and I won’t go outside of that. Suppose I see that as a way to avoid problem gambling. Although I don’t think I could be tempted into problem gambling, I just figure that having money set aside that I can afford to lose and not overstepping that limit is a safeguard. (Kate, Receptionist, 21 years old)

The participants understood problem gambling as spending more than they planned at the venue and gambling more than their financial circumstances permit. Simply, that gambling must stop when disposable funds run out. However, this line of thinking may lead to a decision to gamble more if they have abundant funds available for recreational gambling. Without internal control, control of gambling based on external constraints and availability of funds may not be effective and could lead to problems in the future.

I won’t go too far but…. I won’t stop now (Tristan, Apprentice Carpenter, 18 years old)

You want to go out, go have a good time, do it; you want to gamble, do it, just not at the expense of other people. (Peter, Unemployed, 19 years old)

Participants compared themselves to how they envisioned a problem gambler might think and positioned themselves as the polar opposite. They also attempted to distance themselves further by questioning the intelligence of problem gamblers in a sarcastic tone.

Like actually carrying a rabbit’s foot around with me saying oh this is my lucky charm, this is going to bring me luck! (Laughs) (Theo, Sales Assistant, 24 years old)

Everyone has their different theories, like if you put $20 in and cash it then just put little bits in at a time. Or if you put a $50 in, and you stick a card in the push button, so it just presses it continuously never letting it go, it’s disturbing. (Lisa, University Student, 20 years old)

In the discourse of the participants, the contrast between ‘us’ and ‘them’ was possibly exaggerated to assure that the interviewees had no problem with gambling. They might have also felt judged by discussing the stigmatised issue of gambling. Thus, it was quite important for them to distinguish themselves from problem gamblers and suggest that problem gambling stems from purely individual problems. Hence, it is not gambling that can be harmful but the way “other gamblers” engage in it. This “it’s not related to me” discourse suggests that the participants may not fully understand the risk factors with problem gambling, which starts as a leisure activity. Further, the discursive repertoire overlooks the action and cognitions associated with problem gambling. Most of the erroneous gambling-related beliefs (EGRB), even as a simple example, were yet to surface in the consciousness of the participants. Therefore, whether or not their gambling was problematic at this time was unable to be determined by them because the yardstick by which they compared their actions did not measure their behaviour or consider EGRB’s.

I generally try to stick to the racing where there is some sort of skill involved. I just find it’s a bit more hands on a bit more enjoyable than pushing a button over and over again. (Kate, Receptionist, 21 years old)

Therefore, if they do not understand how problem gambling relates to them or how erroneous gambling-related beliefs could set in, in a different setting under different circumstances, their awareness at this basic level may not fully protect them from the risk of developing a problem with gambling. Another issue may be that the participants’ strong focus on their current life stage may prevent them from confronting the issue of gambling and its potential impact on later stages of life. However, the participants’ confidence to act as mature young adults who are rational, realistic and mature gamblers who have learnt from others’ mistakes may be a protective factor as their discourse also included statements of basic understanding of how gambling works and a resolve that they participate only as a leisure activity.

I’ve won $150, and it’s like this is great, then every time you go in there you expect it. Until you just have to grow up and realise and just go Jesus I’ve spent that much money and I could have put it toward, I could have gone away on a holiday with my girlfriend or whatever. (Peter, Unemployed, 19 years old)

This discursive repertoire presents young people as individuals who are aware of the danger of problem gambling but position themselves in opposition because they had learned from others’ mistakes and knew the risks of gambling. They exhibit a security and satisfaction that they understand gambling, which may contrast with reality.

## Discussion

This study indicates that the way in which young adults understand gambling and acknowledge their involvement is complex. Their discourse reveals how they feel positioned, position themselves and position those around them in relation to gambling. The three dominant repertoires ‘Culture not self,’ ‘If it makes you happy,’ and ‘No problem here!’ reflect the introduction of gambling through family tradition, gambling as a means of socialising, and as a way to describe themselves as rational, intelligent decision makers separating themselves from problem gamblers. In different ways, all three repertoires acknowledge that gambling could lead to problems and include behavioural description of high risk behaviours. However, the image of risk is not well grounded as it describes behavioural characteristics of problem gambling in a different generation, behaviours they believe are qualitatively different from their own. The participants’ assumption and self-definition of social recreational gambling, which is the opposite end of problem gambling, could be problematic. This assumption of “immunity” could delay their help seeking if they develop problems in later years (Sullivan 2001).

Although the participants dismissed the notion of luck because of its known association with problem gambling (e.g., lucky charms), the knowledge and familiarity regarding the gambling forms may lead to higher levels illusion of control (Moore and Ohtsuka 1999a, b). Setting limits was a good sign, but they admitted they would gamble more if they could afford to and their repertoires rely on others to define gambling harms (e.g., it is alright to gamble so long as it does not hurt anybody). These misunderstandings could reflect their age and naiveté or a form of rebellion as a process of establishing their own identity. Personal disengagement from harm minimisation warnings in discourse repertoires shows only partial understanding of the harm minimisation message. The young recreational gamblers do not believe that harm minimisation messages apply to all gamblers—not just for “them,” problem gamblers. Since harm minimisation warnings are more effective when they are personal, relevant and immediate (Mills 2002), the future challenge is how to make harm minimisation messages relevant to young recreational gamblers.

Further, the repertoires also included approval of risk-taking behaviour, which is a common personality trait found in this age group (Gupta and Derevensky 1998). Social norms in favour of gambling, i.e., the family and social acceptance of gambling, was evident in the repertoires. The theme of gambling as social activities and their needs to please family and friends while seeking acceptance from a social group of friends was most evident in the testimony. The current findings are consistent with research on youth gambling showing the influence of social norms for young gamblers (Derevensky et al. 1996).

The three repertoires contradict each other in that they use their language to both alleviate personal responsibility for gambling and take credit for practicing responsible gambling. More emphasis is placed on the former as gambling is a social act that defines their identity as a member of a family and community where social norms in support of gambling normalise and encourage gambling. In such environments, readdressing their understanding of responsible gambling is difficult without the involvement of significant others and the community. The young adults, although as independent as they would like to be, develop and define their idea of self in a relational term in the context of a network of relationships with significant others. Therefore, any intervention would have to target families and friendship groups and even recreational clubs where many young people’s friendships are made.

The current research on the discourse of young adult gamblers in Australia has many theoretical implications to help understand how gambling forms part of a culture and is maintained as a social activity that binds the members of social groups. The three interpretive repertoires of young adult gambling capture the subjective meanings and cultural values of the informants and have presented rich qualitative information in the context of the Australian urban culture. Despite young adults constituting large numbers of gaming venue clients, gaming venue managers are least sympathetic towards young gamblers, more specifically young men, concerning the provision of responsible gambling (O’Mahony and Ohtsuka 2015). Young gamblers’ strong self-identification which distances themselves from problem gambling further prevents them from receiving necessary due attention from the gaming staff, thereby increasing gambling risk.

The current investigation has made a significant contribution to a growing body of knowledge on subjective meanings of gambling, which informs the interpretation, understanding, and values of people who gamble. Furthermore, harm minimisation strategies targeting young recreational gamblers could be fine tuned to increase their understanding. The current investigation also highlighted the importance of social interactions, context, and perceived shared cultural values for young recreational gamblers.

Social norm is a powerful predictor of intention and human behaviour (see Theory of Reasoned Action, Ajzen and Fishbein 1975, 1980; Ajzen and Madden 1986; Theory of Planned Behaviour, Ajzen 1991) including gambling behaviour (Martin et al. 2010; Moore and Ohtsuka 1997, 1999a, b). This qualitative enquiry also highlights the role of the social norm in which the family introduction to gambling is positively remembered. Not surprisingly, the family introduction to gambling was reported as a predictor of gambling behaviour (Ladouceur et al. 1994; Ladouceur and Mireault 1988). Longitudinal studies on gambling “career” highlight the importance of social influence on trajectories of gambling in a UK community where gambling forms part of core activities (Reith and Dobbie 2011, 2012, 2013). In these cases, social norms and perceived subjective norms are predominantly positive towards gambling. For the participants in the current study, gambling was associated with family activities in the past and was regarded as a positive recreational activity.

Perhaps, gambling is regarded in such positive light for some participants to the extent that problem gambling is condemned not so much because of its negative impact on the life of problem gamblers but to bring disrepute to otherwise wholesome entertainment and social activity. While their participation in gambling is responsible, lack of information and insight in detecting signs of problem gambling may be problematic for some in the future. Another clear identity described in their discourse was the belief that young adults have less financial obligations and a strong sense of self-control. This optimistic assumption that they are not at risk of problem gambling may be problematic if their circumstances change in the future.

Cultural factors were often investigated only for gamblers from culturally and linguistically diverse (CALD) communities (e.g., Ohtsuka and Ohtsuka 2010; Ohtsuka 2013; Stevens and Golebiowska 2013). The current research suggests that the “mainstream” group (e.g., Anglo Australians) also have a distinct “culture” with cultural traditions, values and beliefs. Further, the “mainstream” culture is not necessarily a uniform mass but consists of diverse subgroups with their own different identities and subcultural values. Such is the case of young adult Australians who socialise at pubs—a segment or subgroup within the mainstream Australian gamblers.

In-depth interviews and discourse analysis reveal that the participants share common threads that weave a tapestry of the subculture which may not be well documented if due attention is directed to their cultural imports. The current study has demonstrated the relationships between gambling as shared social activities, family, and a sense of belongingness and identities of the young Australian gamblers.

In a sense, the current findings compliment Ohtsuka’s (2013) qualitative research on electronic gaming machine players in suburban clubs from CALD backgrounds. Through the discourse analysis, the current investigation has explored subjective views of young adults who gamble in Australian suburban clubs. Although their demographics may vary, through their social network and their family backgrounds, they shared their stories using similar discourse repertoires allowing a glimpse into subjective worlds of a sub-group with similar outlooks concerning gambling participation, values and understanding of gambling.

### Implication for future research

This study indicates that the way in which young adults understand gambling and acknowledge their own involvement is a complex and difficult issue. All the participants were involved in some form of gambling on a weekly basis, yet gambling did not consciously occupy a prominent place in their life. The three repertoires identified ‘Culture not self,’ ‘If it makes you happy,’ and ‘No problem here!’ are similar in some ways but differentiate in others. Although three repertoires acknowledge that gambling can lead to problem gambling, the image of problem gambling is somewhat general, and they felt little relevance to their self-definition as young recreational gamblers. Dismissive views on erroneous gambling-related beliefs would further prevent them from gaining insight on how their confidence in self-control may conspire to support beliefs in luck. Young gamblers feel more comfortable and ‘know’ some forms of gambling better than others, but familiarity breeds illusion of control. While emphasising that they, unlike problem gamblers, always set limits on spending, they would gamble more if they could afford, and it is ok so long as gambling does not hurt anybody. Ambivalent views cannot be dismissed as naiveté. It is also a form of rebellion against what young gamblers perceive as social pressure for conformity. The self-definitions of young gamblers need further investigation because these should be taken into account to make harm minimisation messages truly relevant to young gamblers.

The three repertoires include inherent contradictions in their attempt to both alleviate personal responsibility for gambling and take credit for what they consider to be responsible gambling. More emphasis is placed on the former as gambling is characterised as a social act partly defining their identity as a member of a family and community who they feel normalises and encourages this activity. As such, readdressing their understanding of responsible gambling is unworkable without the cooperation of significant others and the community as their idea of self is relational and situated in a network of relationships with other selves. Therefore, any intervention would have to target families and friendship groups and even recreational clubs where many young people’s friendships are made.

### Limitations

Discourse analysis focuses on the subjective values and specific ways in which the participants use the language to understand their gambling in the social context. Subjective meanings may entail idiosyncratic views. Since gambling is specific to a jurisdiction, the repertoires of young gamblers may reflect their demographics (e.g., English-speaking white Australians with secondary education or higher who gamble socially at clubs). Repertoires may not be shared by young adult gamblers from culturally and linguistically diverse (CALD) backgrounds. Hence, generalisation beyond demographics and age groups may require caution.

## Conclusion

To summarise, discourse analysis of young gamblers shows that gambling originated from family culture and tradition. It formed a focal point of social interaction among family members and friends. Hence, young adult gamblers hold positive attitude and regard it with affection. They also see themselves as belonging to a privileged phase of their life when the freedom to gamble is legitimised as an act of rebellion and a reward for their effort to live up to family expectations. They are aware that their lifecycle allows them to engage in little risk-taking for fun. Although young gamblers are aware of problem gambling risks, their understanding is stymied by the dissociation between themselves and the older problem gamblers from different socioeconomic strata.

The current study demonstrated the potential benefits of qualitative research focused on discourse repertoires to explore subjective meanings and values of gamblers. The emphasis on cultural values and tacit agreements share common approaches to cultural enquiries on gambling. Beyond examining specific meanings associated with gambling, an important aim was to establish discourse analysis as a possible theoretical framework for studying gambling and in doing so, allowing researchers to look beyond the quantifying extent of young adult gambling to an in-depth understanding of how gambling is understood among young adult gamblers. While further study is needed to establish the relationships between discourse and gambling practices, this case study underscores the need for both researchers and policymakers to consider how discourse shapes experiences among young adults.

## Appendix: Interview questions

- How did you come to be here tonight?
  - Do you like this place and hotels/pubs in general?
- Where would you normally go to socialise?
  - How do you spend your leisure time?
- Have you ever gambled?
  - Do you think that over the course of the evening you will gamble?
    - What makes you think so?
- Do your friends/family gamble?
- Do you remember and can you describe for me, your first gambling experience?
  - E.g. playing poker with parents? Handheld electronic gambling devices?
- Do you have any particular memories or stories about gambling that you could share?
  - Describe your best and experience/worst experience
- How would you describe your usual gambling experience?
  - Routine/night out, winning/losing
- Do you ever participate in the lottery or those TV/radio programs where a call costs 55c to $1 and you register your name/number to win money?
- Do you believe in luck?
  - Have you ever experienced luck? Describe
- Do you believe in fate or the idea that everyone has a destiny?
- Are you in any way religious?
- Do you think there is such a thing as pure chance?
- Which games do you generally play?
  - Do you think that there is an element of skill involved?
- How do you increase your chances of winning?
  - Is winning important? What is (important)?

## Footnotes

1 Young adults are different from adolescents who have more time at hand but little money to gamble.

2 The participants’ view of their environment and social circles.
